# Transcultural Diabetes Nutrition Algorithm: A Malaysian Application

**DOI:** 10.1155/2013/679396

**Published:** 2013-12-09

**Authors:** Zanariah Hussein, Osama Hamdy, Yook Chin Chia, Shueh Lin Lim, Santha Kumari Natkunam, Husni Hussain, Ming Yeong Tan, Ridzoni Sulaiman, Barakatun Nisak, Winnie Siew Swee Chee, Albert Marchetti, Refaat A. Hegazi, Jeffrey I. Mechanick

**Affiliations:** ^1^Department of Medicine, Hospital Putrajaya, Pusat Pentadbiran Kerajaan Persekutuan, Presint 7, 62250 Putrajaya, Malaysia; ^2^Division of Endocrinology, Diabetes and Metabolism, Joslin Diabetes Center, Harvard Medical School, Boston, MA 02215, USA; ^3^Department of Medicine, University Malaya Medical Centre, Kuala Lumpur, Malaysia; ^4^Department of Medicine, Hospital Pulau Pinang, Penang, Malaysia; ^5^Department of Medicine, Hospital Tengku Ampuan Rahimah, Selangor, Malaysia; ^6^Family Medicine, Putrajaya Health Clinic, Putrajaya, Malaysia; ^7^Department of Health Care, International Medical University, Kuala Lumpur, Malaysia; ^8^Department of Dietetics and Food Services, Hospital Kuala Lumpur, Kuala Lumpur, Malaysia; ^9^Department of Nutrition and Dietetics, University Putra Malaysia, Selangor, Malaysia; ^10^Department of Nutrition and Dietetics, International Medical University, Kuala Lumpur, Malaysia; ^11^Preventive Medicine and Community Health, University of Medicine and Dentistry of New Jersey, Newark, NJ 07101, USA; ^12^Abbott Nutrition, Columbus, OH 43219, USA; ^13^Division of Endocrinology, Diabetes, and Bone Disease, Icahn School of Medicine at Mount Sinai, New York, NY 10029, USA

## Abstract

Glycemic control among patients with prediabetes and type 2 diabetes mellitus (T2D) in Malaysia is suboptimal, especially after the continuous worsening over the past decade. Improved glycemic control may be achieved through a comprehensive management strategy that includes medical nutrition therapy (MNT). Evidence-based recommendations for diabetes-specific therapeutic diets are available internationally. However, Asian patients with T2D, including Malaysians, have unique disease characteristics and risk factors, as well as cultural and lifestyle dissimilarities, which may render international guidelines and recommendations less applicable and/or difficult to implement. With these thoughts in mind, a transcultural Diabetes Nutrition Algorithm (tDNA) was developed by an international task force of diabetes and nutrition experts through the restructuring of international guidelines for the nutritional management of prediabetes and T2D to account for cultural differences in lifestyle, diet, and genetic factors. The initial evidence-based global tDNA template was designed for simplicity, flexibility, and cultural modification. This paper reports the Malaysian adaptation of the tDNA, which takes into account the epidemiologic, physiologic, cultural, and lifestyle factors unique to Malaysia, as well as the local guidelines recommendations.

## 1. Introduction 

Globally, the prevalence of prediabetes and type 2 diabetes (T2D) is increasing as a consequence of social, epidemiologic, and demographic shifts, such as population aging and urbanization [1, 2]. The majority of people with these conditions now live in low- and middle-income countries, including many Asian nations, where substantial increases in incidence rates are anticipated by the year 2030 [2]. According to the fourth Malaysian National Health and Morbidity Survey (NHMS IV) carried out in 2011, the prevalence of T2D in Malaysian adults ≥30 years of age had risen to 20.8%, affecting an estimated 2.8 million individuals [3] as compared with the third National Health and Morbidity Survey (NHMS III), which reported a prevalence of 14.9% in 2006 [4]. The heterogeneous nature of Asian populations gives rise to unique T2D features. For example, Asians tend to develop T2D at a lower body mass index (BMI), at younger age, and with a lower waist circumference than Caucasians [5, 6], and their course of illness is punctuated with earlier chronic complications [7–9] and frequent postprandial hyperglycemia [10]. These and other clinical features must be recognized and factored into lifestyle recommendations in order to tailor management to individual needs and improve the effectiveness of preventive and therapeutic efforts at the primary care level.

## 2. Methods and Materials 

The universal tDNA template for patients with prediabetes and T2D was established by an international task force of experts during a two-year process that included planning and developmental meetings, evidence collection and review, consensus building, and algorithm construction and face validation [11]. The initial global template was designed for simplicity, flexibility, and cultural modification. A comparable process was used by an appointed Malaysian task force to adapt the algorithm to meet the needs of practitioners and patients in Malaysia. The regional version emerged through the modification of general tDNA recommendations to account for cultural, lifestyle, food, diet, and genetic differences that exist among the Malaysian people.

### 2.1. Perspectives Unique to Malaysia

Among the major ethnic groups in Malaysia, Indians (24.9% in 2011 and 19.9% in 2006) had the highest prevalence of T2D, followed by Malays (16.9% in 2011 and 11.9% in 2006) and Chinese (13.8% in 2011 and 11.4% in 2006) [3, 4]. These epidemiologic differences could be due to the genetic makeup, diet, and cultural variants among these major ethnic groups.

The overall prevalence of abdominal obesity in Malaysia, measured by waist circumference, has been reported between 55.6% and 57.4% [13, 14]. Epidemiologic studies investigating abdominal obesity in Malaysia have consistently shown an ethnic trend similar to that seen in T2D with prevalence being highest among Indians (65.5–68.8%), followed by Malays (55.1–60.6%), Chinese (49.5–51.1%), and other indigenous groups (44.9–48.3%) [13, 14]. The prevalence of abdominal obesity is increased among patients with T2D and is observed in 75% of T2D patients in Malaysia. Moreover, in the DiabCare Malaysia 2008 study, the most recent study in an ongoing initiative to monitor diabetes control in Malaysia, undesirable waist circumference was reported in a higher proportion of women (≥80 cm in 89.4%) than men (≥90 cm in 73.7%) with T2D [15]. The study patients with T2D, 72% of whom were obese, had a mean BMI of 27.8 kg/m^2^.

Glycemic control in Malaysia continues to deteriorate despite initiatives by the Ministry of Health to increase awareness and also expanded accessibility of glycosylated hemoglobin (A1c) testing across the country. The DiabCare Malaysia 2008 study reported a mean A1c of 8.66%, compared with 8.0% [16] in 2003, a mean fasting glucose of 8.0 mmol/L, and an elevated mean postprandial glucose of 12.7 mmol/L in Malaysians with T2D. Furthermore, only 22% of the patients achieved the glycemic target of A1c <7%, the lowest rate since 1998 [15]. Data from the online registry database Adult Diabetes Control and Management (ADCM) revealed ethnic differences in glycemic control and complication profiles among Malaysians. Chinese patients had the lowest mean A1c levels, while Malaysian Indians had the highest [17].

Only 16.4% of the Malaysian patients adhere to the dietary regimen provided by dietitians [20]. Interestingly, patients were found to adhere to the advice of “eat lots of food high in dietary fiber such as vegetables or oats” but found it difficult to eat five or more servings of fruits and vegetables per day. Self-care practices among the majority of patients with suboptimal glycemic control are obviously inadequate. A large proportion of Malaysian T2D patients consume four or more meals a day and more than two carbohydrate portions per snack [21].

**Figure 1:**
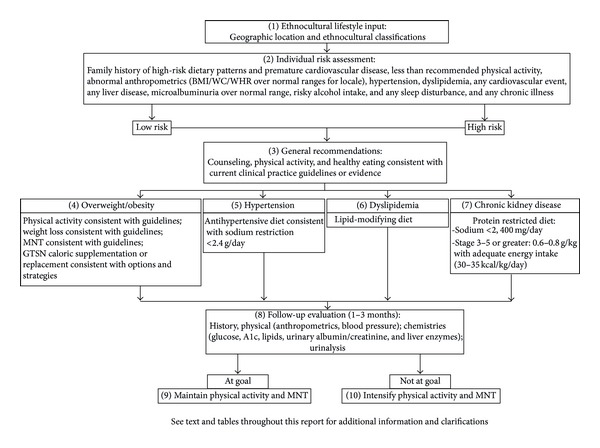
Transcultural Diabetes Nutrition Algorithm (tDNA): Malaysian application.

The current Malaysia Clinical Practice Guidelines (CPGs) for the management of T2D contain recommendations without any specific reference to glycemia-targeted specialized nutrition (GTSN), that is, oral nutritional products that facilitate glycemic control and may be used as meal and/or snack replacements or supplements as part of the medical nutrition therapy (MNT) [18]. With the increasing prevalence of prediabetes and T2D and the continued deterioration of glycemic control among patients in Malaysia, there is a clear need for a simple MNT algorithmic decision-making tool to address these issues. This paper summarizes the Malaysian adaptation of the universal tDNA template [11]. See Figure 1. Specific Southeast Asian and Asian Indian tDNA versions have also been published [22, 23].

## 3. Results: Transcultural Factors for Malaysia

### 3.1. Assessment of Body Composition and Risk of Disease Progression

The World Health Organization (WHO) Western Pacific Regional Office and the International Diabetes Foundation (IDF) define overweight and obesity in Asians as BMI greater than 23 kg/m^2^ and 25 kg/m^2^, respectively [24]. Lower cutoff values are required for Asian populations because Asians generally have a higher percentage of intra-abdominal fat compared with Caucasians of the same age, sex, and BMI [25]. Furthermore, Asian populations have higher cardiovascular and T2D risk factors than Caucasians at any BMI level [25, 26], thereby highlighting the rationale for defining Asian-specific cutoff values for anthropometric measures.

The Malaysian CPG for the management of obesity categorizes overweight as BMI of 23.0–27.4 kg/m^2^ and obesity as BMI of 27.5 kg/m^2^ and above [28]. Waist circumference cutoff values for abdominal obesity are 90 cm for men and 80 cm for women [24]. Similarly, these cutoff values are also found in the CPG for the management of T2D in Malaysia [12] and are used as the standard throughout this paper.

### 3.2. Physical Activity in T2D Management

Physical activity and exercise have been shown to lower blood glucose levels, improve glucose and insulin utilization, and improve carbohydrate metabolism [29, 30]. Benefits of physical activity have been demonstrated in both Caucasian and Asian patients with T2D [31–34]. The Malaysian CPG for the management of T2D recommends physical activity as an integral feature in every stage of T2D management [12]. These recommendations are echoed in the Malaysian tDNA application (Table 1).

### 3.3. MNT and Weight Loss in T2D Management

MNT plays an integral role in T2D management and indeed is recommended by the American Diabetes Association as an important component of individual weight loss programs for T2D patients [35]. The benefits of MNT on glycemic control in Asians with prediabetes and T2D have been demonstrated in clinical trials [36–39]. On-site registered dietitian-led management of MNT has been shown to improve glycemic control in poorly-managed patients with T2D in primary care clinics in Taiwan. Patients with A1c levels ≥7% who received on-site diabetic self-management education had significantly greater improvements in fasting plasma glucose and A1c levels after one year than control subjects or subjects with A1c levels <7% [36]. A lifestyle intervention that includes MNT was found to be effective in preventing or delaying the development of T2D in middle-aged Japanese patients with impaired glucose tolerance [40, 41].

The Malaysian Dietitians' Association (MDA) has formed an expert committee, comprising dietitians from primary care, hospitals, and academia, to compose MNT recommendations for T2D. The first version was published in 2005 [42] and updated in 2013 [43]. Building on the MNT guidelines recommended by the MDA, the Malaysian CPG for the management of T2D, and taking into consideration similar Malaysian CPGs for hypertension and dyslipidemia, this panel recommends the nutritional considerations outlined in Table 2 [12, 18, 19].

Weight loss is an important therapeutic objective for T2D patients to reduce insulin resistance. Moderate weight loss of just 5–10% of body weight in patients with T2D has been shown to decrease insulin resistance and improve other metabolic risk factors [38, 44, 45]. GTSN formulae are a component of MNT that contain nutrients to facilitate weight management and glycemic control. These formulae are available in Malaysia and may be utilized with nutritional counseling as meal and/or snack replacements for overweight and obese patients and those with suboptimal glycemic control, including persons with high insulin requirements. These formulae are also indicated as a supplementary nutrition for patients with diabetes and acute concurrent illness who are unable to maintain optimal nutrition due to reduced appetite and calorie intake. Recommendations for the use of meal replacements will be incorporated in the revised MNT guidelines from the MDA.

### 3.4. Nutritional Management of Patients with Concomitant Hypertension, Dyslipidemia, and/or Chronic Kidney Disease (CKD)

Data from the ADCM's online registry database showed that as many as 57% of the Malaysian patients with T2D experience concomitant hypertension [46]. Among the ethnic groups in Malaysia, more Malay patients (62.3%) have concomitant hypertension than Chinese (19.6%) or Indian (17.0%) patients. In patients with T2D, hypertension is defined as blood pressure >130/80 mmHg on two readings 2-3 weeks apart [12]. Pharmacotherapy for hypertension should be initiated in patients with T2D when the blood pressure is persistently >130 mmHg systolic and/or >80 mmHg diastolic [12]. For patients with concomitant hypertension, salt intake should be restricted to <6 g/day (sodium 2 g) [18].

The ADCM also revealed that as many as 38% of the patients with T2D in Malaysia suffer from concomitant dyslipidemia [47]. Malays were more likely to have uncontrolled low-density lipoprotein cholesterol (LDL-C) and triglycerides compared with Chinese and Indians; however, Indians were twice as likely to have inadequate high-density lipoprotein cholesterol compared with Malays [47]. A recent study that investigated the ethnic differences in lipid metabolism among Malaysian patients with T2D demonstrated that Malays had significantly higher serum levels of glycoxidation and lipoxidation products compared with those of Chinese and Indian patients [48]. For T2D patients with dyslipidemia, lifestyle modification focusing on the reduction of saturated fat (<7% of total calories), trans fat (avoid), and cholesterol (<200 mg/day) intake has been recommended [12, 19]. In accordance with the Malaysian CPG for dyslipidemia, patients over the age of 40 without overt cardiovascular disease (CVD) should be treated with lipid lowering drugs, regardless of the baseline LDL-C levels, while all patients with overt CVD, irrespective of age, should be treated with lipid lowering drugs [19].

For T2D patients with concomitant CKD, limited protein intake and daily sodium <2400 mg are recommended. For those with CKD stages 3–5, daily protein should be limited to 0.6–0.8 g/kg in a diet with adequate energy intake (30–35 kcal/kg/day) [49].

## 4. Conclusions

The following recommendations, statements, figures, tables, and graphs represent the conclusions of the Malaysian transcultural Diabetes Nutrition Algorithm (tDNA) task force and constitute the current Malaysian tDNA application, which accommodates local differences in lifestyle, foods, and customs and incorporates established local Clinical Practice Guidelines (CPGs) to meet the needs and preferences of type 2 diabetes (T2D) patients in Malaysia.

*Recommendation 1*. Medical nutrition therapy (MNT) is an integral component of the management of T2D and must be prioritized in view of poor glycemic control among patients in Malaysia. Individualized care plans are essential in order to increase adherence and compliance with MNT recommendations.

*Recommendation 2*. Personalized nutrition counseling by a dietitian is recommended and should be individualized according to personal nutritional needs, concomitant disease, severity of T2D, cultural preferences, and patient cooperation. If access to a dietitian is not possible, all newly diagnosed patients should receive basic nutrition and dietary counseling from either doctors or diabetes educators.

*Recommendation 3*. Values for body mass index (BMI) cutoffs in the Malaysian CPGs are recommended for use in the Malaysian tDNA.

*Recommendation 4*. The Malaysian CPG for the management of T2D recommends physical activity as an integral feature in every stage of T2D management [12]. These recommendations are adopted in the Malaysian tDNA (Table 1). 

*Recommendation 5*. Overweight and obese individuals should achieve a weight loss of 5–10% of the initial body weight over a 6-month period (Table 2). 

*Recommendation 6*. The nutritional recommendations outlined in Table 2 (adapted from the Malaysian Dietitians' Association's MNT guidelines and the Malaysian CPG for the management of T2D) should be implemented as part of the Malaysian tDNA. 

*Recommendation 7*. Patients with T2D and concomitant hypertension should limit salt intake to <6 g/day (sodium 2 g). Those with concomitant chronic kidney disease (CKD) should limit protein intake, especially those with CKD stages 3–5 (daily protein of 0.6–0.8 g/kg with adequate energy intake of 30–35 kcal/kg/day). 

*Recommendation 8*. Lifestyle modification focusing on the reduction of saturated fat (<7% of total calories) and cholesterol (<200 mg/day), as well as the avoidance of trans-fat, is recommended for patients with T2D and concomitant dyslipidemia. 

*Recommendation 9*. The use of meal replacements should be based on clinical judgment and individual assessment. For patients who are overweight, meal and/or snack replacements are recommended as part of meal plans to reduce total calorie intake (Table 3). For patients of normal weight with uncontrolled T2D, 1-2 servings of a GTSN formula per day, incorporated into a meal plan as meal or snack replacement, are recommended. For underweight individuals, 1–3 servings of a GTSN formula per day are recommended as supplementation based on the clinical judgment and individual assessment of desired rate of weight gain and clinical tolerance. 

*Recommendation 10*. To provide support and motivate patients to comply with MNT, monthly follow-ups are recommended for patients with poorly-controlled T2D and for those who are at high risk of complications. For patients with well-controlled T2D, regular follow-up every 3 months is recommended.

## Figures and Tables

**Table 1:** Physical activity guidelines for the management of type 2 diabetes^a^ [12].

	Frequency	Exercise 5 days a week with no more than 2 consecutive days without physical exercise
All patients	Intensity and type	(i) Moderate-intensity activities include walking down stairs, cycling, fast walking, doing heavy laundry, ballroom dancing (slow), noncompetitive badminton, and low-impact aerobics (ii) Vigorous activities include jogging, climbing stairs, football, squash, tennis, swimming, jumping rope, and basketball
	Duration	150 min per week of moderate-intensity aerobic physical activity and/or at least 90 min per week of vigorous aerobic physical activity

Overweight or obese patients (BMI > 23)		Gradually increase physical activity to 60–90 minutes daily for long-term major weight loss

BMI: body mass index.

^a^Patients should be assessed for complications that may preclude vigorous exercise. Age and previous physical activity level should be considered.

**Table 2:** Nutrition guidelines for the management of type 2 diabetes [12, 18, 19].

Calories	For overweight and obese individuals, a reduced calorie diet of 20–25 kcal/kg body weight is recommended to achieve a weight loss of 5–10% of initial body weight over a 6-month period
Carbohydrate	45–60% daily energy intake
Protein	15–20% daily energy intake
Fat	25–35% daily energy intake
Saturated fat	Less than 7% of total calories
Cholesterol	Less than 200 mg/day
Fiber*	20–30 g/day
Sodium	<2,400 mg/day

*Should be derived predominantly from foods rich in complex carbohydrates including grains (especially whole grains), fruits and vegetables.

**Table 3:** Glycemia-targeted specialized nutrition (GTSN) for the management of prediabetes and type 2 diabetes.

Overweight (BMI > 23 kg/m^2^) or obese (BMI > 27.5 kg/m^2^)		Use meal and/or snack replacements^a^ as part of a meal plan to reduce total calorie intake (i) Calorie reduction of 500–1000 calories per day (to lose 0.5–1.0 kg per week), using 1-2 servings of a GTSN formula^b^ to replace 250–500 calories from meals (ii) Reassess every 1–3 months

Normal weight (BMI 18–23 kg/m^2^)	Controlled diabetes (A1c ≤ 6.5%^c^)	The use of meal replacements should be based on clinical judgment and individual assessment^d^
	Uncontrolled diabetes (A1c > 6.5%^c^)	Use 1-2 servings of a GTSN formula per day to be incorporated into a meal plan

Underweight (BMI < 18 kg/m^2^)		Use 1–3 servings of a GTSN formula per day as supplementation based on clinical judgment and individual assessment of desired rate of weight gain and clinical tolerance

BMI: body mass index; A1c: glycosylated hemoglobin; GTSN: glycemia-targeted specialized nutrition.

Recommendations were rated and assigned numerical and alphabetical descriptors according to levels of scientific substantiation provided by the 2010 American Association of Clinical Endocrinologists protocol for the development of Clinical Practice Guidelines [27].

^a^Meal and snack replacements are nutritional products used as replacement for meals or snacks to replace calories in the diet. It is suggested that products used should meet the American Diabetes Association nutritional guidelines.

^b^Glycemia-targeted specialized nutrition formulas are complete and balanced products with at least 200 calories per serving used as part of a meal plan to help control calorie intake and achieve glycemic control.

^c^Glycemic (A1c) targets should be individualized for each patient based on local CPGs.

^d^To avoid hypoglycemia or postprandial hyperglycemia, individuals who may have muscle mass and/or function loss and/or micronutrient deficiency may benefit from a nutrition supplement. Individuals who need support with weight maintenance and/or a healthy meal plan could benefit from meal replacement.
